# Supplementary material for: Single-cell analysis of two severe COVID-19 patients reveals a monocyte-associated and tocilizumab-responding cytokine storm
Source: Nat Commun. 2020 Aug 6;11:3924. doi: 10.1038/s41467-020-17834-w (PMC7413381; doi:10.1038/s41467-020-17834-w)
Supplement: Supplementary file 1 — Supplementary Information [file 41467_2020_17834_MOESM1_ESM.pdf]

Supplementary Information

Single-cell analysis of two severe COVID-19 patients reveals a monocyte-associated and tocilizumab-responding cytokine storm

Guo, et al.

Supplementary Fig. 1

a

Batch name	Captured cells	Median genes per cell	Median UMIs per cell	Cells passed QC
P1-day1-rep1	2591	840	2068	1511
P1-day1-rep2	2081	862	2100	1257
P1-day5-rep1	3129	682	1850	2160
P1-day5-rep2	3851	691	1895	2699
P2-day1	2280	914	2067	1576
P2-day5	1488	810	1909	1027
P2-day7	3332	977	2238	3009

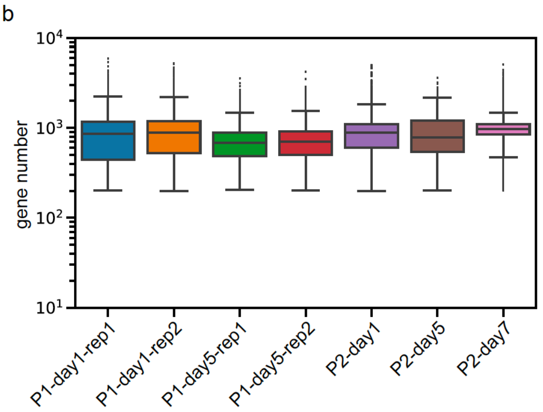

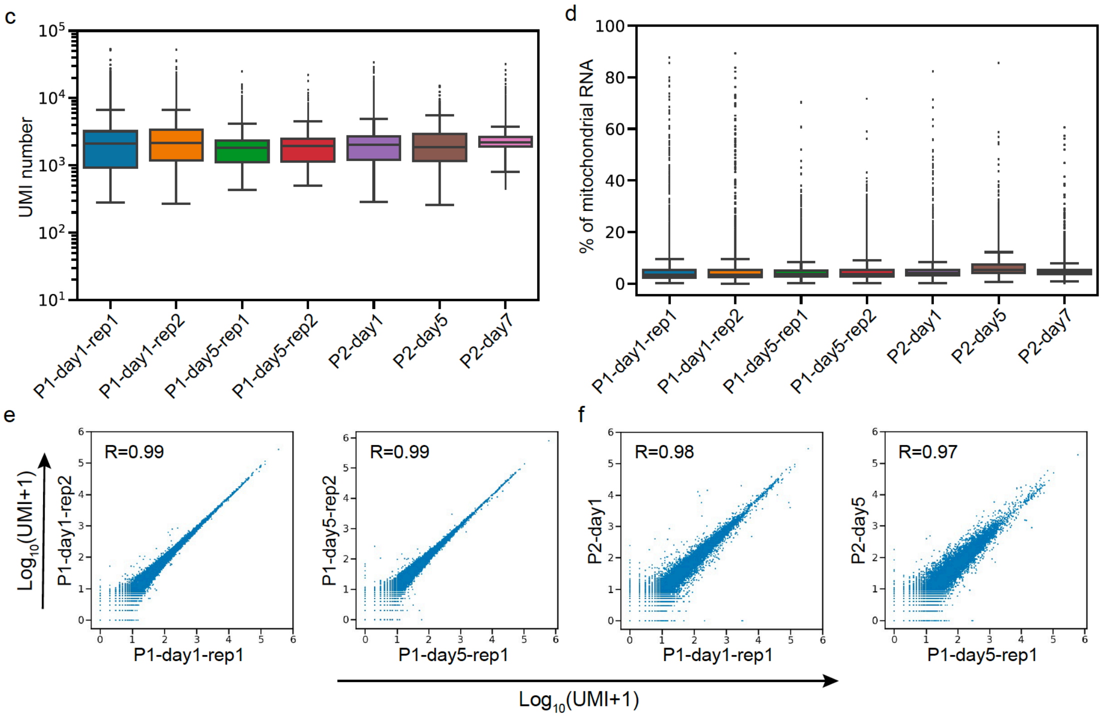

Supplementary Figure 1 | Quality control of single-cell data for PBMC samples from severe COVID-19 patients. a, Summary of captured cells, median genes per cell, median UMIs per cell, and the number of cells that passed quality control (QC) in distinct batches of single-cell data from severe COVID-19 patients. **b-d,** Box plots showing the gene number **(b)**, UMI number **(c)**, and percentage of mitochondrial RNA **(d)** in distinct batches of single-cell data from severe COVID-19 patients. **e, f,** Aggregated scRNA-seq one-to-one reproducibility plots for technical replicates **(e)** and biological replicates **(f)**. The correlation (R) represents the Pearson correlation across all genes. Box-whisker plot; the lower whisker is the lowest value greater than the 25% quantile minus 1.5 times the interquartile range (IQR), the lower hinge is the 25% quantile, the middle is the median, the upper hinge is the 75% quantile and the upper whisker is the largest value less than the 75% quantile plus 1.5 times the IQR.

Supplementary Fig. 2

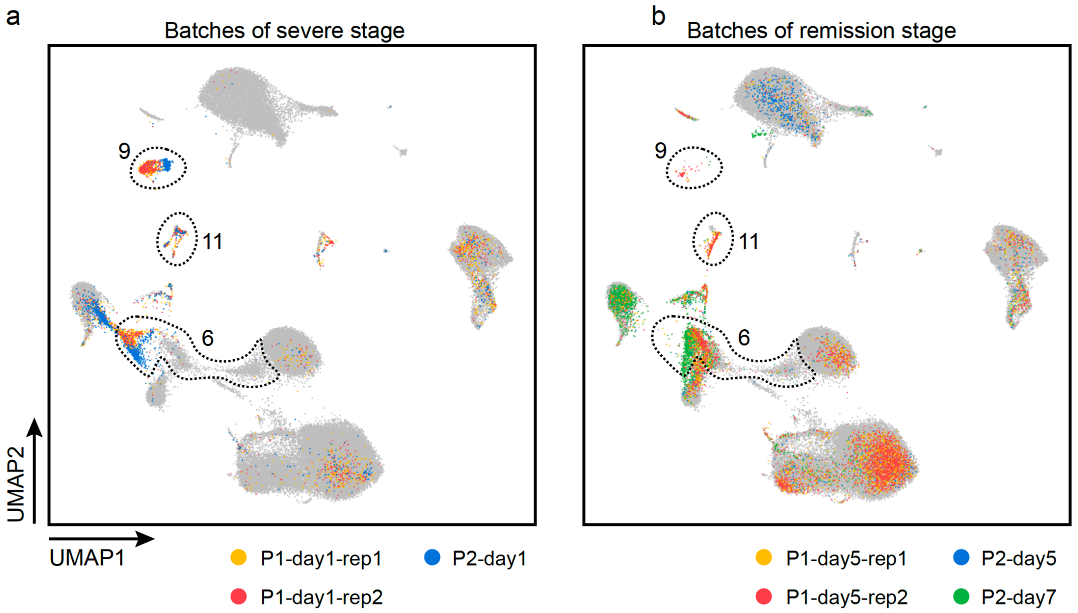

Supplementary Figure 2 | Single-cell transcriptomes of PBMCs from patient P1 or P2 at each time point. a, UMAP plot showing single-cell transcriptomes from patient P1 and P2 on day 1. **b,** UMAP plot showing single-cell transcriptomes from patient P1 at day 5 and P2 on day 5 and day 7.

Supplementary Fig. 3

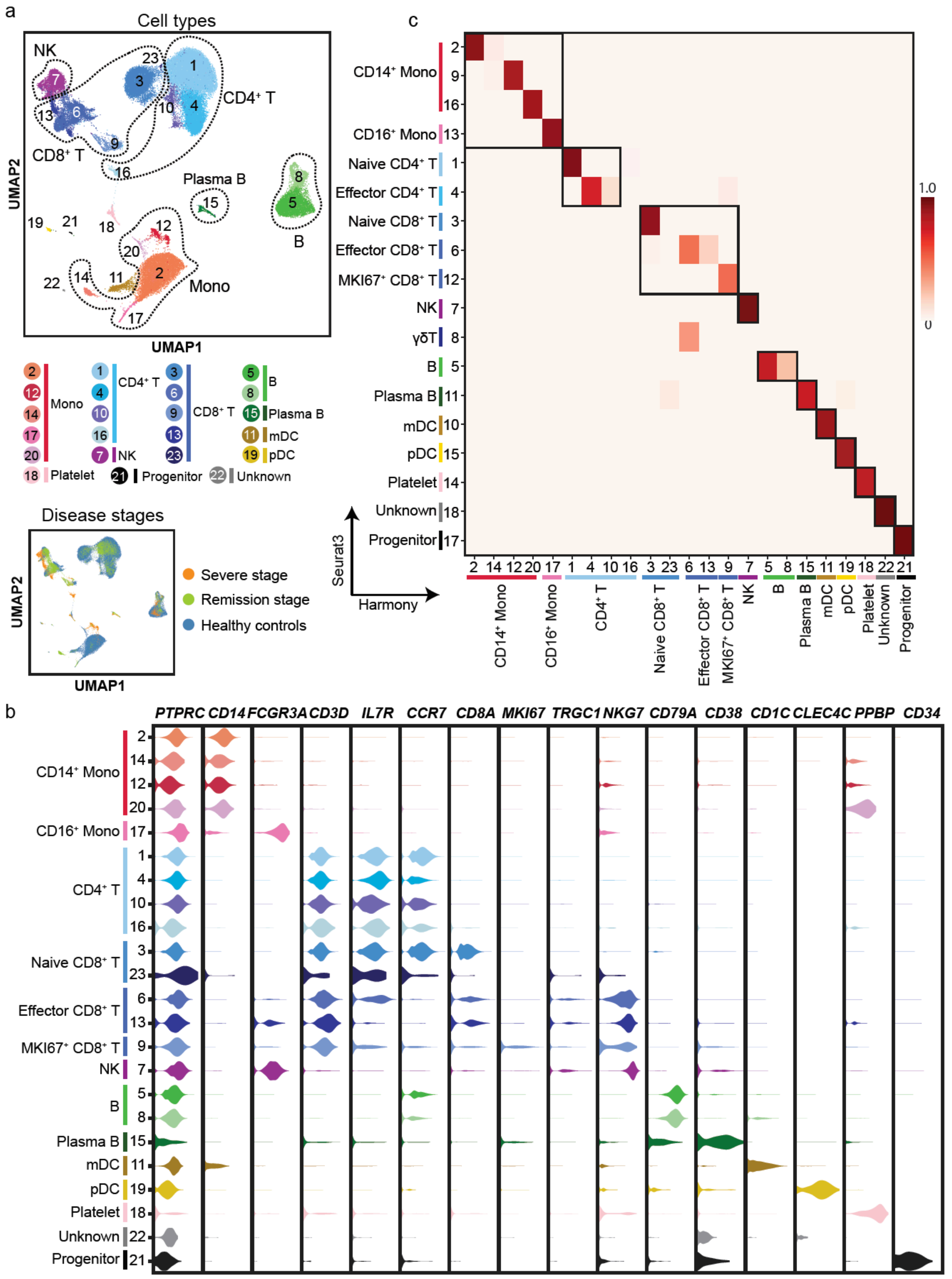

Supplementary Figure 3 | Single-cell profiling of peripheral immune cells in severe COVID-19 integrated with healthy controls using Harmony. a, UMAP representations of single-cell transcriptomes of 69,237 PBMCs integrated by Harmony. Cells are colour-coded by clusters and disease states (see legend for key). Mono, monocyte; NK, natural killer cell; mDC, myeloid dendritic cell; pDC, plasmacytoid dendritic cell. **b,** Violin plots of selected marker genes (upper row) for multiple cell subpopulations. The left column presents the cell subtypes as identified based on combinations of marker genes. **c**, Jaccard similarities between the cell clusters with the integration processed by Seurat (version 3.1.4) and with the integration processed by Harmony.

Supplementary Fig. 4

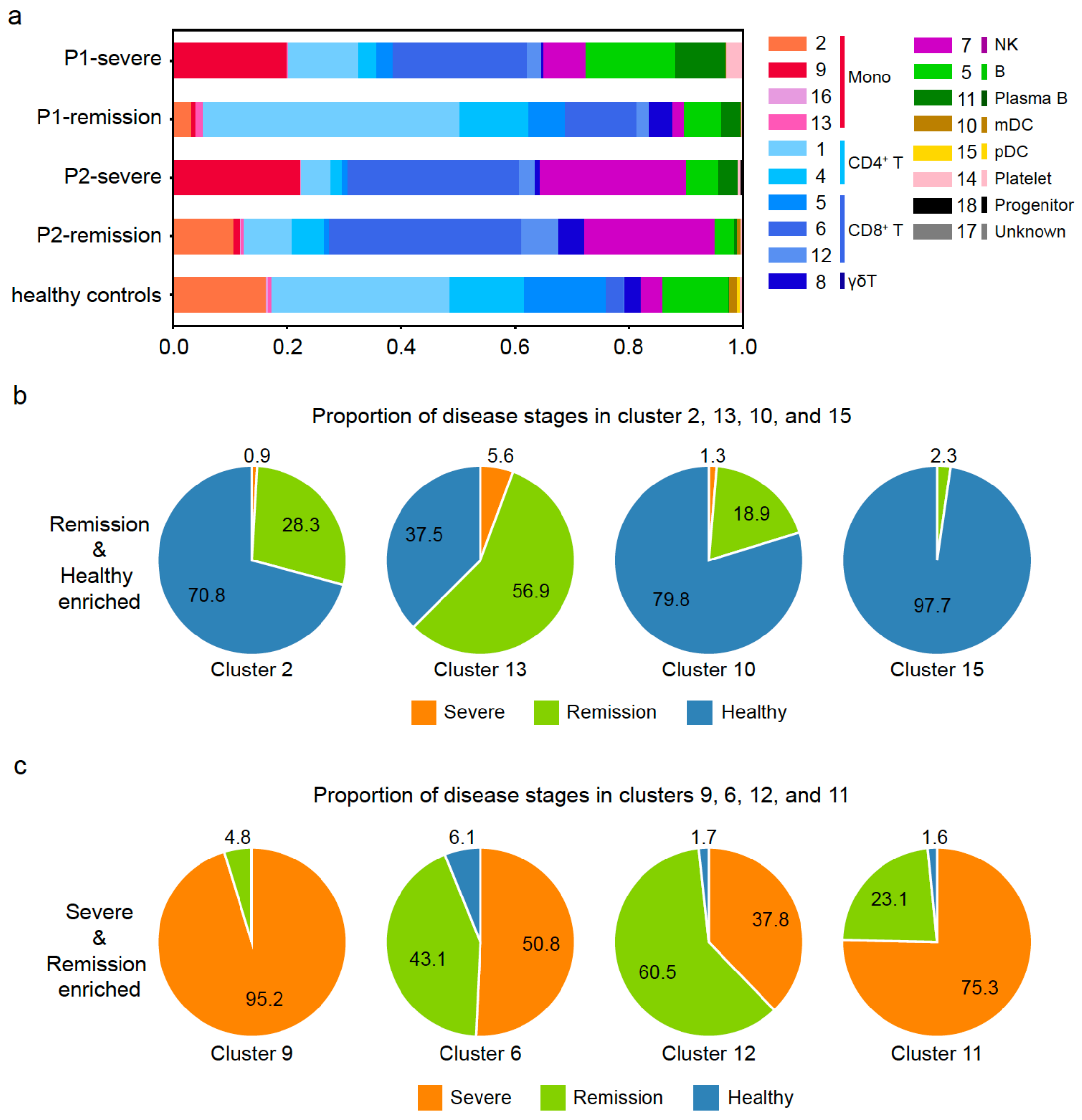

Supplementary Figure 4 | The composition of cell clusters identified in the integrated single-cell transcriptomes of PBMCs from the severe and remission stages, and in healthy control individuals. a, Bar chart showing the percentage of cell clusters in the severe and remission stages and in healthy control individuals. Source data are provided as a Source Data file. **b**, Pie chart showing the proportion of cells from each disease state in selected cell clusters (clusters 2, 13, 10, 15), which were present in remission-stage patients and in healthy controls, but not in severe-stage patients. **c**, Pie chart showing the proportion of cells from each disease state in selected cell clusters (clusters 9, 6, 12, 11), which were present in severe and remission stages but not in healthy controls.

Supplementary Fig. 5

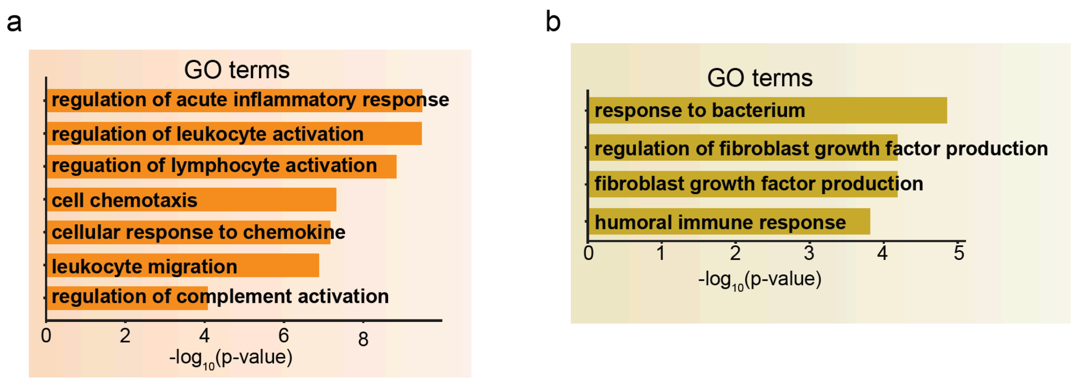

Supplementary Figure 5 | GO terms enriched among DEGs highly expressed in monocyte at the severe stage or at severe and remission stages. a, b, Bar plots of enriched GO terms of genes highly expressed in monocytes at the severe stage **(a)** or at the severe and remission stages **(b)**. *P* values were calculated using a hypergeometric test and the Benjamini-Hochberg correction algorithm (i.e. multi-test adjustments) in Metascape.

Supplementary Fig. 6

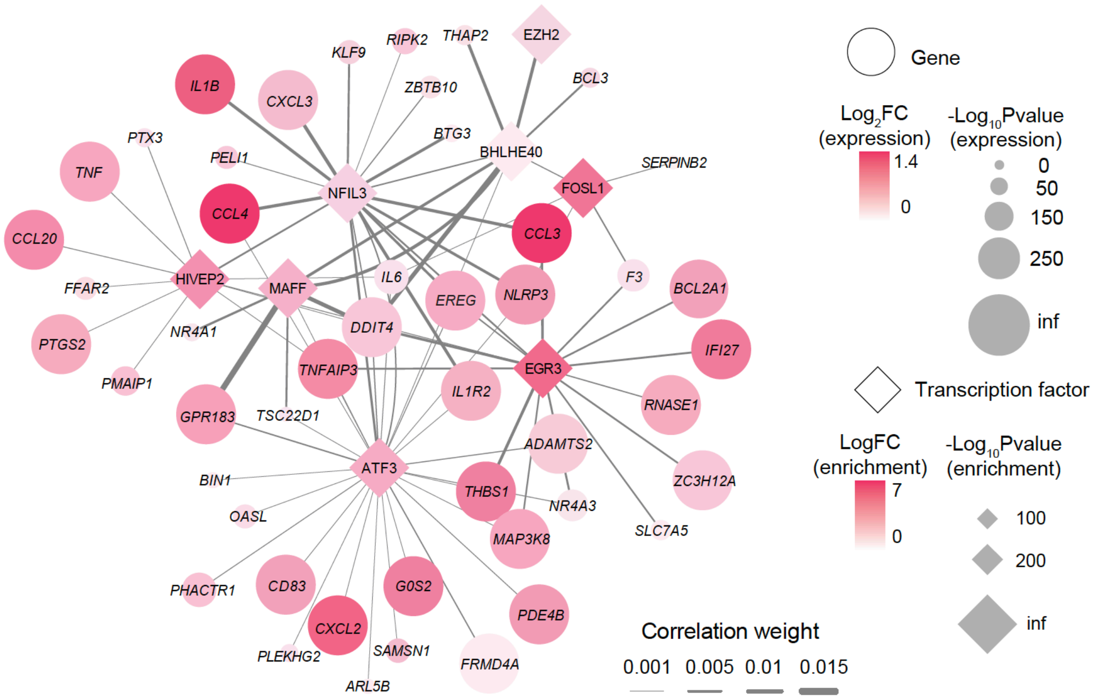

Supplementary Figure 6 | Severe-stage-specific monocyte regulatory network predicted by SCENIC. Transcription factors are shown as rectangles; their target genes are shown as circles. Student's t-test. *P* values were calculated using two-sided Student's t-test.

Supplementary Fig. 7

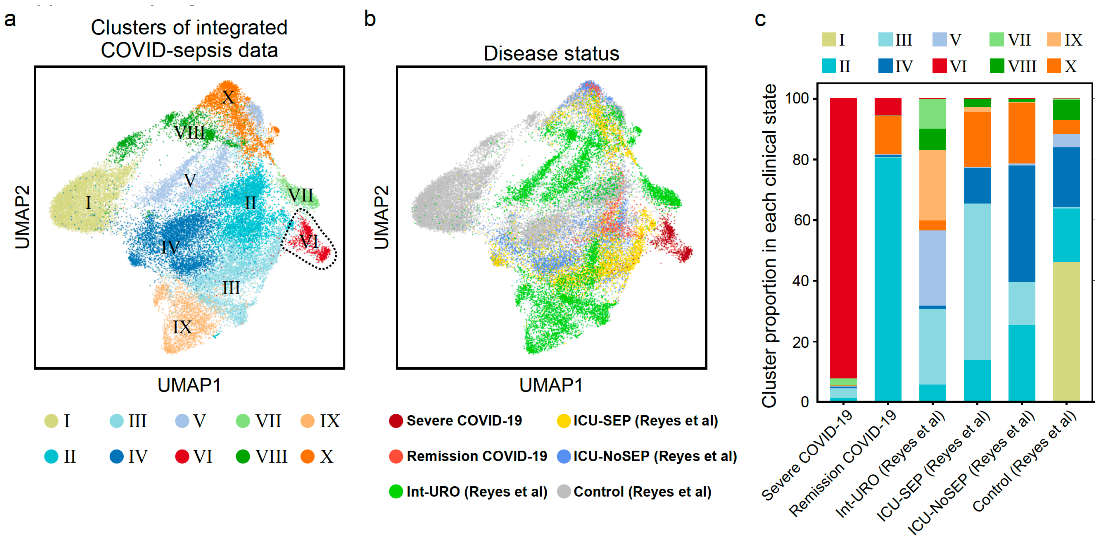

Supplementary Figure 7 | Integrated single-cell transcriptome analysis from patients with sepsis and our COVID-19 patients. a, b, UMAP representations of integrated single-cell transcriptomes from patients with sepsis at mild stage (Int-URO, n = 7)[1], patients with sepsis at severe stage (ICU-SEP, n = 8)[1], critically ill patients without sepsis (ICU-NoSEP, n = 7)[1], healthy controls from outside our study (Control, n = 19)[1], and our COVID-19 patients (Severe COVID-19 and remission COVID-19). Cells are colour-coded by clusters **(a)**, disease states **(b)**. **c**, Bar chart showing the proportion of cell clusters in (**a**) in each disease state. Source data are provided as a Source Data file.

Supplementary Fig. 8

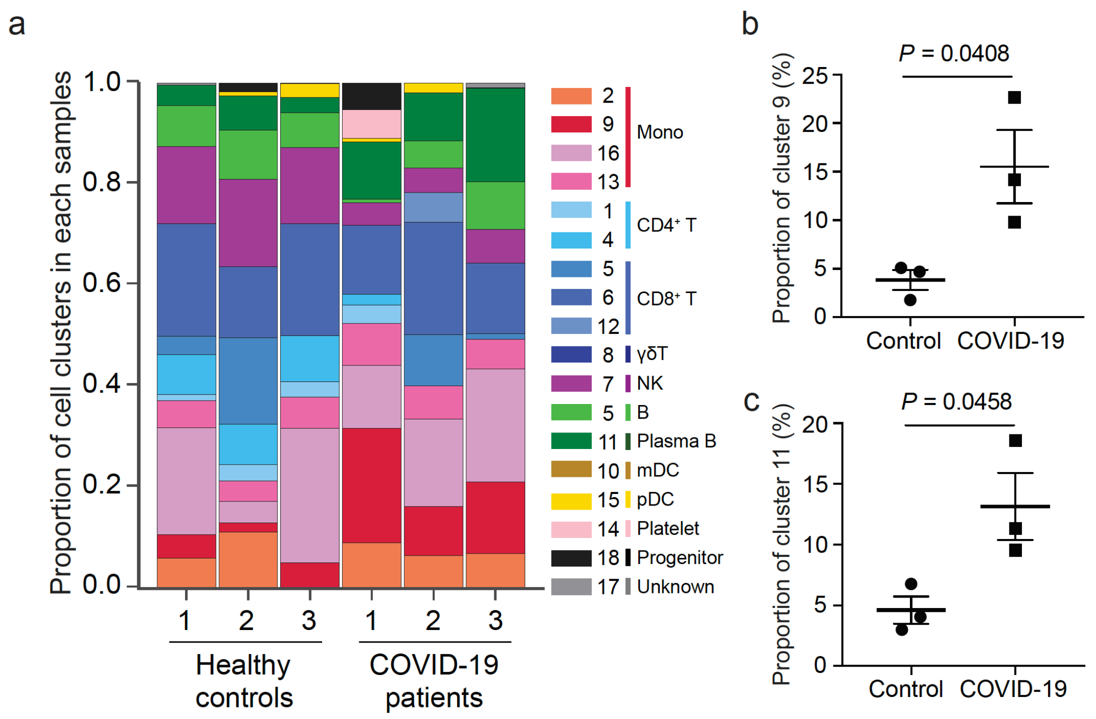

Supplementary Figure 8 | The composition of cell clusters identified in our single-cell analysis in a bulk RNA-seq from three severe COVID-19 patients and healthy controls. a, Bar chart showing an estimation of the composition of each cell cluster of PBMCs deconvoluted from bulk RNA-seq data from three COVID-19 patients and healthy controls[2]. Source data are provided as a Source Data file. **b, c**, Dot plot showing the percentage of severe stage-specific monocytes (cluster 9, **b**) and plasma B cells (cluster 11, **c**) in COVID-19 patients and healthy controls, deconvoluted from bulk RNA-seq. *P* values were calculated using two-sided Student's t-test. Error bars denote standard error of mean (SEM), and the individual measurement values (each from n = 3 biologically independent samples) are shown as black dots. Source data are provided as a Source Data file.

Reference

1 Reyes, M. *et al.* An immune-cell signature of bacterial sepsis. *Nat Med*, doi:10.1038/s41591-020-0752-4 (2020).

2 Xiong, Y. *et al.* Transcriptomic characteristics of bronchoalveolar lavage fluid and peripheral blood mononuclear cells in COVID-19 patients. *Emerg Microbes Infect* **9**, 761-770, doi:10.1080/22221751.2020.1747363 (2020).
