# Supplementary material for: Single-cell analysis of two severe COVID-19 patients reveals a monocyte-associated and tocilizumab-responding cytokine storm
Source: Nat Commun. 2020 Aug 6;11:3924. doi: 10.1038/s41467-020-17834-w (PMC7413381; doi:10.1038/s41467-020-17834-w)
Supplement: Supplementary file 3 — Reporting Summary [file 41467_2020_17834_MOESM3_ESM.pdf]

nature research

Corresponding author(s): NCOMMS-20-15446A (NCOMMS-20-15446-T) Kun Qu

Last updated by author(s): Jul 17, 2020

Reporting Summary

Nature Research wishes to improve the reproducibility of the work that we publish. This form provides structure for consistency and transparency in reporting. For further information on Nature Research policies, see our Editorial Policies and the Editorial Policy Checklist.

Statistics

For all statistical analyses, confirm that the following items are present in the figure legend, table legend, main text, or Methods section.

n/a	Confirmed	
☐	☒	The exact sample size (*n*) for each experimental group/condition, given as a discrete number and unit of measurement
☐	☒	A statement on whether measurements were taken from distinct samples or whether the same sample was measured repeatedly
☐	☒	The statistical test(s) used AND whether they are one- or two-sided<br>*Only common tests should be described solely by name; describe more complex techniques in the Methods section.*
☒	☐	A description of all covariates tested
☒	☐	A description of any assumptions or corrections, such as tests of normality and adjustment for multiple comparisons
☐	☒	A full description of the statistical parameters including central tendency (e.g. means) or other basic estimates (e.g. regression coefficient) AND variation (e.g. standard deviation) or associated estimates of uncertainty (e.g. confidence intervals)
☐	☒	For null hypothesis testing, the test statistic (e.g. *F*, *t*, *r*) with confidence intervals, effect sizes, degrees of freedom and *P* value noted<br>*Give P values as exact values whenever suitable.*
☒	☐	For Bayesian analysis, information on the choice of priors and Markov chain Monte Carlo settings
☒	☐	For hierarchical and complex designs, identification of the appropriate level for tests and full reporting of outcomes
☒	☐	Estimates of effect sizes (e.g. Cohen's *d*, Pearson's *r*), indicating how they were calculated

Our web collection on statistics for biologists contains articles on many of the points above.

Software and code

Policy information about availability of computer code

Data collection	1) Genome: All the human data was aligned to the hg38 assembly.<br>2) Annotation: Human gene annotation was integrated from Refseq and UCSC.<br>3) Data integration: (1) All the scRNA-seq data of PBMCs from COVID-19 patients was generated by us; (2) scRNA-seq data for PBMCs from healthy controls was from 10X Genomics official website [https://support.10xgenomics.com/single-cell-gene-expression/datasets/3.1.0/5k_pbmc_NGSC3_aggr]; (3) Additional scRNA-seq data sets from patients with sepsis and critically ill patients without sepsis were downloaded from the Broad Institute Single Cell Portal [https://singlecell.broadinstitute.org/single_cell]: SCP548 (patients PBMCs); (4) Additional bulk RNA-seq datasets of PBMCs from 3 severe COVID-19 patients and 3 healthy controls were downloaded from Genome Sequence Archive in BIG Data Center [https://bigd.big.ac.cn/] under the accession number: CRA002390.
Data analysis	We uses CellRanger (version 3.1.0) and aligned the raw sequencing reads to the GRCh38 human reference genome. We used Seurat (version 3.1.4) for cell filtering, count matrix normalization, cell clustering, and dimension reduction (PCA and UMAP). We applied Seurat (version 3.1.4) and Harmony (version 1.0) to integrate the sequencing data of PBMCs from the patients and healthy controls. For differential genes analysis we used Scipy 1.0.0 in Python 3.6.8. Genes involved in selected GO terms were downloaded from the GSEA-MSigDB website (https:// www.gsea-msigdb.org/gsea/msigdb/index.jsp). For ligand-receptor interaction analysis we uploaded the scRNA-seq count matrix to the CellphoneDB website (https://www.cellphonedb.org/explore-sc-rna-seq). Enriched GO terms of the DEG groups were estimated using the Metascape website (https://metascape.org/gp/index.html#/main/step1) with the default parameters. SCENIC (version 1.1.2) and RcisTarget database were used to build the gene regulatory network of monocytes, and we applied Cytoscape (version 3.8.0) to construct the connection map for the network. The bulk RNA-seq data was deconvoluted by using AutoGeneS (version 1.0.3)

For manuscripts utilizing custom algorithms or software that are central to the research but not yet described in published literature, software must be made available to editors and reviewers. We strongly encourage code deposition in a community repository (e.g. GitHub). See the Nature Research guidelines for submitting code & software for further information.

Data

Policy information about availability of data

All manuscripts must include a data availability statement. This statement should provide the following information, where applicable:

- Accession codes, unique identifiers, or web links for publicly available datasets
- A list of figures that have associated raw data
- A description of any restrictions on data availability

The scRNA-seq data of PBMCs from the 2 severe COVID-19 patients can be obtained from the Gene Expression Omnibus (GEO) database, and the accession number is GSE150861 [https://www.ncbi.nlm.nih.gov/geo/query/acc.cgi?acc=GSE150861]. We also used published datasets as controls or comparable data, including (1) the scRNA-seq data of PBMCs from 2 healthy donors downloaded from the 10X Genomics official website [https://support.10xgenomics.com/single-cell-gene-expression/datasets/3.1.0/5k_pbmc_NGSC3_aggr]; (2) the scRNA-seq data of PBMCs from 22 sepsis patients and 19 related controls, which is available on the Institute Single Cell Portal [https://singlecell.broadinstitute.org/single_cell] under accession number SCP548; (3) the bulk RNA-seq data of PBMCs from 3 COVID-19 patients and 3 related controls, which were downloaded from the GSA at the BIG Data Centre under accession number CRA002390 [http://bigd.big.ac.cn/gsa/browse/CRA002390]; and (4) the GRCh38 human reference genome used for the sequencing data alignment, which is available on the 10X Genomics official website [https://support.10xgenomics.com/single-cell-gene-expression/software/downloads/latest]. Source data are provided with this paper.

Field-specific reporting

Please select the one below that is the best fit for your research. If you are not sure, read the appropriate sections before making your selection.

- [x] Life sciences
- [ ] Behavioural & social sciences
- [ ] Ecological, evolutionary & environmental sciences

For a reference copy of the document with all sections, see nature.com/documents/nr-reporting-summary-flat.pdf

Life sciences study design

All studies must disclose on these points even when the disclosure is negative.

Sample size	We collected the blood samples from 2 severe COVID-19 patients (P1, P2) on day 1 and on day 5, and obtained another blood draw from P2 on day 7. Our decision to obtain blood draws from the two patients on day 5 was guided by information from the authors of the study published in PNAS (Xu, X. et. al. 2020), which prompted our decision to consider day 5 of tocilizumab treatment as the beginning of the remission stage. For patient P2, we observed that his SARS-CoV-2 nucleic acid test of a throat swab specimen was still positive on day 5, so we performed another blood draw on day 7 for P2, at which point the throat swab specimen nucleic acid test was negative. We generated 13,239 high-quality transcriptomes for single PBMCs from the 2 severe COVID-19 patients, and then we integrated a total of 68,190 cells comprising the single-cell transcriptomes from the 2 COVID-19 patients and the published single-cell profiles of healthy PBMCs from the 10X official website. The single-cell transcriptomes of PBMCs for each patient examined separately were quite similar and exhibited patterns consistent with our discoveries.
Data exclusions	For cells from COVID-19 patients and healthy donors, we removed cell doublets using Scrublet (https://github.com/AllonKleinLab/scrublet). Then, we filtered low-quality cells using Seurat (version 3.1.4): for cells from COVID-19 patients (P1and P2), we retained cells with detected gene numbers between 500 and 6,000 and less than 10% mitochondrial UMIs; for cells from healthy donors, we retained cells with detected gene numbers between 300 and 5,000 and less than 10% mitochondrial UMIs. All exclusion criteria were pre-established for this data analysis.
Replication	We did scRNAseq on 2 COVID-19 patients at 3 different time points (day 1 and day 5 for patient P1; day 1, day 5, and day 7 for P2) from the severe to the remission stages. Single-cell RNA-seq data from patient P1 got technical replicates on day 1 and day 5. We confirmed the gene expression profiles of most of the identified cell subtypes were quite similar between the biological/technical replicates using the Jaccard index quantification.
Randomization	Not applicable. Samples were segregated based on clinical status (for COVID-19 patients).
Blinding	Non-blinded study. All the researchers involved in the study were aware of the clinical status (for COVID-19 patients).

Reporting for specific materials, systems and methods

We require information from authors about some types of materials, experimental systems and methods used in many studies. Here, indicate whether each material, system or method listed is relevant to your study. If you are not sure if a list item applies to your research, read the appropriate section before selecting a response.

Materials & experimental systems

n/a	Involved in the study
☒	☐ Antibodies
☒	☐ Eukaryotic cell lines
☒	☐ Palaeontology and archaeology
☒	☐ Animals and other organisms
☐	☒ Human research participants
☒	☐ Clinical data
☒	☐ Dual use research of concern

Methods

n/a	Involved in the study
☒	☐ ChIP-seq
☒	☐ Flow cytometry
☒	☐ MRI-based neuroimaging

Human research participants

Policy information about studies involving human research participants

Population characteristics	Patient P1 was a severe patient for his peripheral capillary oxygen saturation (SPO2) <93% without nasal catheter for oxygen. Patient P2 was critical ill for respiratory failure, multiple organ dysfunction (MOD) and SPO2 <93 without nasal catheter for oxygen. Detailed clinical characteristics were summarized in Supplementary Table. 1
Recruitment	The blood samples from the 2 severe COVID-19 patients were collected from the First Affiliated Hospital of University of Science and Technology of China. Our 2 samples were collected with no self-selection bias. We found substantial inter-individual variations in the proportion of NK cells; therefore we have excluded further consideration of NK cells from our manuscript.
Ethics oversight	Ethical approval was obtained from the ethics committee of the First Affiliated Hospital of the University of Science and Technology of China (No. 2020-XG(H)-020).

Note that full information on the approval of the study protocol must also be provided in the manuscript.
