# Supplementary material for: Single-cell analysis of two severe COVID-19 patients reveals a monocyte-associated and tocilizumab-responding cytokine storm
Source: Nat Commun. 2020 Aug 6;11:3924. doi: 10.1038/s41467-020-17834-w (PMC7413381; doi:10.1038/s41467-020-17834-w)
Supplement: Supplementary file 4 — Description of Additional Supplementary Files [file 41467_2020_17834_MOESM4_ESM.pdf]

Description of Additional Supplementary Files

Supplementary Data 1 | Baseline characteristics and laboratory findings for the two COVID-19 patients in this study.

Supplementary Data 2 | Sequencing data quality.

Supplementary Data 3 | Differentially expressed genes (DEGs) of different disease stages of the monocytes.

Supplementary Data 4 | GO terms enriched among DEGs in different disease stages of the monocytes.

Supplementary Data 5 | Sets of genes entailed in the enriched GO terms from Figures 2e and 2f.

Supplementary Data 6 | Interactions of cytokines and receptors in different disease stages, predicted using CellphoneDB.

Supplementary Data 7 | Drugs targeting cytokines or cytokine receptors.

Supplementary Data 8 | DEGs of different disease stages of effector $CD8^+$ T cells.

Supplementary Data 9 | GO terms enriched among DEGs in different disease stages of the effector $CD8^+$ T cells.

Supplementary Data 10 | Set of genes entailed in the enriched GO terms from Figures 4h and 4i.
